# Posttranslational Regulation of Inflammasomes, Its Potential as Biomarkers and in the Identification of Novel Drugs Targets

**DOI:** 10.3389/fcell.2022.887533

**Published:** 2022-06-21

**Authors:** Sambit K. Nanda, Stefan Vollmer, Ana B. Perez-Oliva

**Affiliations:** ^1^ Bioscience Immunology, Research and Early Development, Respiratory and Immunology (R&I), Gaithersburg, MD, United States; ^2^ Bioscience COPD/IPF, Research and Early Development, Respiratory and Immunology (R&I), Gothenburg, Sweden; ^3^ Instituto Murciano de Investigación Biosanitaria (IMIB)-Arrixaca, Murcia, Spain; ^4^ Centro de Investigación Biomédica en Red de Enfermedades Raras (CIBERER), Instituto de Salud Carlos III, Madrid, Spain

**Keywords:** inflammasome, phosphorylation, ubiquitylation, SUMOylation, acetylation, ADP-ribosylation, prenylation, citrullination

## Abstract

In this review, we have summarized classical post-translational modifications (PTMs) such as phosphorylation, ubiquitylation, and SUMOylation of the different components of one of the most studied NLRP3, and other emerging inflammasomes. We will highlight how the discovery of these modifications have provided mechanistic insight into the biology, function, and regulation of these multiprotein complexes not only in the context of the innate immune system but also in adaptive immunity, hematopoiesis, bone marrow transplantation, as well and their role in human diseases. We have also collected available information concerning less-studied modifications such as acetylation, ADP-ribosylation, nitrosylation, prenylation, citrullination, and emphasized their relevance in the regulation of inflammasome complex formation. We have described disease-associated mutations affecting PTMs of inflammasome components. Finally, we have discussed how a deeper understanding of different PTMs can help the development of biomarkers and identification of novel drug targets to treat diseases caused by the malfunctioning of inflammasomes.

## 1 Introduction

Microbial detection is achieved through germline-encoded pattern-recognition receptors (PRRs) that survey both the extracellular and intracellular spaces for pathogens-associated molecular patterns (PAMPs) (Broz and Dixit, 2016). These PRRs include Toll-like receptor (TLR), C-type lectin receptor (CLR), nucleotide-binding oligomerization (NOD)-like receptor (NLR), and RIG-I-like receptor (RLR) families, which detect a diverse array of PAMPs. Members of NLR family, which includes NOD-, LRR- and pyrin domain-containing 1 (NLRP1), NLRP3, NLRP6, NLRP7, NOD-, LRR-and CARD-containing 4 (NLRC4), and related family members such as pyrin as well as Absent in melanoma 2 (AIM2), form a multiprotein complex called the inflammasomes (Zheng et al., 2020). In addition to the PAMPs, some of the PRRs can also be activated by endogenous host products that have similarities to their microbial counterparts. These host products, which are termed damage-associate molecular patterns (DAMP), are generated as a result of metabolic by-products or released upon tissue damage. Some of the inflammasome sensors, such as AIM2 (sensing dsDNA), and NIAPs (sensing flagellin to activate NLRC4), have been shown to activate the inflammasome pathway by directly engaging with their cognate receptor, but several other agonists indirectly lead to inflammasome assembly by perturbing cell homeostasis. The alterations in the cellular homeostasis, termed as homeostasis-altering molecular processes (HAMP), may explain the promiscuity of NLRP3 and pyrin inflammasome, which can be activated by a diverse array of unrelated stimuli (Liston and Masters, 2017).

Recognition of the canonical inflammasome agonists results in sensor activation, its oligomerization, and in most of the cases recruitment of an adaptor protein ASC (also known as PYCARD), which consists of two death-fold domains: a pyrin domain (PYD) and a caspase recruitment domain (CARD). These domains allow ASC to bridge the upstream inflammasome sensor molecule to Caspase-1. Formation of the inflammasome complex turns an inactive Caspase-1 into an active protease, which cleaves pro-interleukin (IL) 1 and pro-IL18 into their bioactive form (Broz and Dixit, 2016). The non-canonical inflammasome is activated following detection of lipopolysaccharide (LPS), a component of Gram-negative bacteria, in the cytosol of cells, resulting in activation of inflammatory caspase 4 and caspase 5 in human cells, and caspase 11 in mouse cells (Kayagaki et al., 2011). These inflammatory caspases cleave the pore-forming protein called gasdermin D (GSDMD) to release the inhibitory carboxy (C)-terminus of GSDMD, allowing active GSDMD amino (N)-terminus to bind to the cell membrane to induce a highly inflammatory form of programmed cell death called pyroptosis (Kayagaki et al., 2015). The pore formation activity of the N-terminal GSDMD causes cell swelling, preventing replication of intracellular pathogens, and is required for the release of the bioactive form of the IL-1 family of cytokines into the extracellular space. IL-1 family of cytokines creates a highly inflammatory environment and leads to the recruitment of inflammatory cells such as neutrophils and natural killer (NK) cells (Aglietti and Dueber, 2017). Therefore, inflammasomes play a crucial role in host defense against pathogens, but aberrant inflammasome activation is linked to the development of autoimmune, metabolic, cardiovascular, respiratory and neurodegenerative diseases as well as cancer (Guo et al., 2015). Tight control of inflammasome assembly and signaling is critical to ensure selectivity and certainty, allowing the immune system to initiate antimicrobial and inflammatory responses while avoiding excessive tissue damage.

**FIGURE 1:**
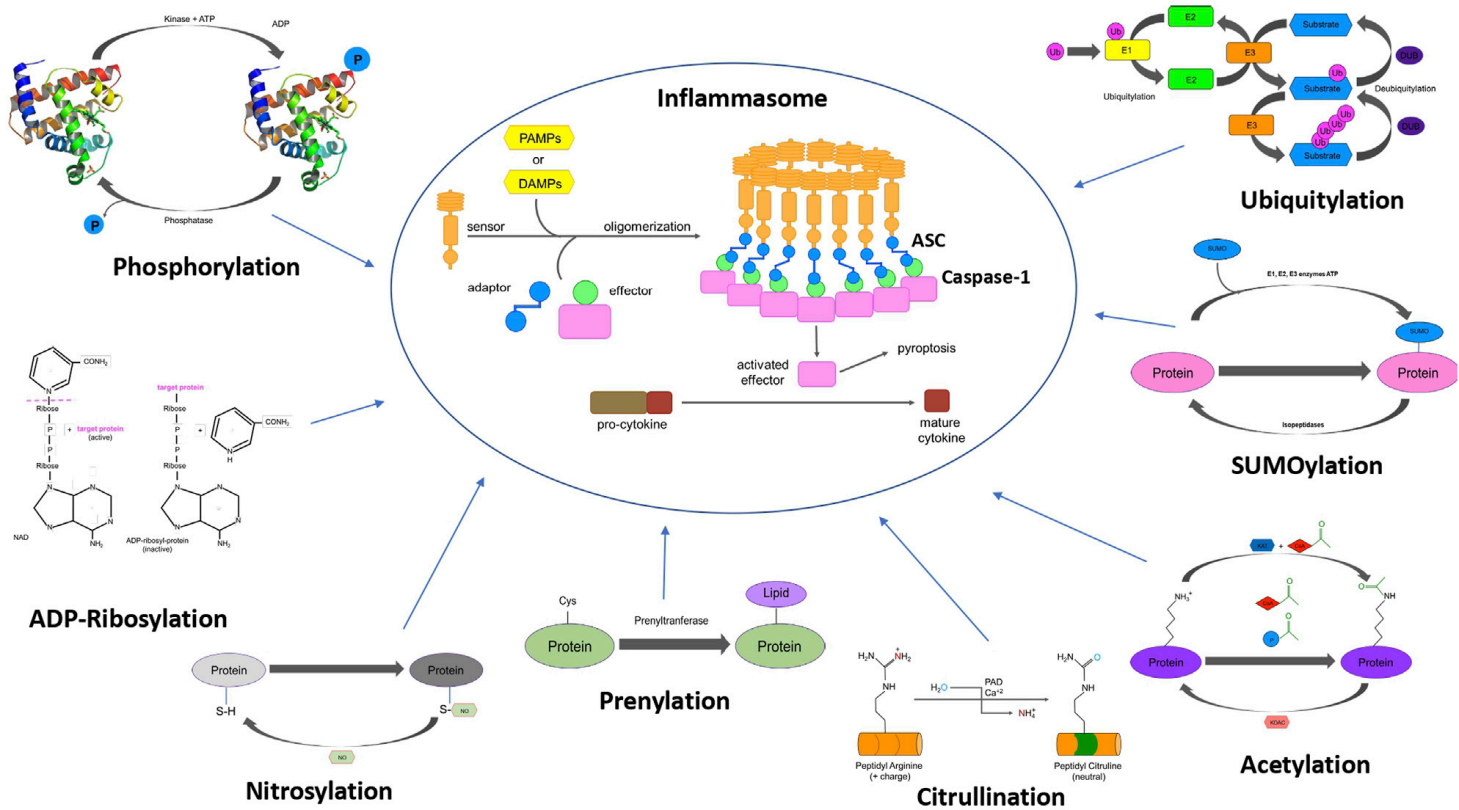
Schematic representation of the PTMs associated with different inflammasome components. The figure shows assembly of inflammasome complex (centre). Different post-translational modifications that modulate inflammasome function such as phosphorylation, ubiquitylation, SUMOylation, acetylation, nitrosylation, ADP-ribosylation, citrullination, and prenylation.

To treat diseases caused by the overactivation of inflammasome and achieve specificity, elucidation of detailed inflammasome signaling is necessary. Despite the involvement of inflammasome signaling in various disease pathologies, not much is known about the biochemical and molecular basis of inflammasome signaling. As an example, the NLRP3 inflammasome activation requires two signals, signal 1 activated by the engagement of a TLR agonist, such as LPS (a TLR4 agonist), and stimulation of signal 2 can be achieved by a wide variety of molecules including extracellular ATP, nigericin, and various components of bacteria and viruses (Broz and Dixit, 2016), but signaling cascades that these two stimuli trigger and promiscuity of NLRP3 inflammasome is not fully understood. It was believed that stimulation of cells with signal 1 for several hours is necessary to transcriptionally upregulate NLRP3 expression, which is needed for caspase 1 activation. This paradigm has changed since recent studies have demonstrated rapid inflammasome activation by co-stimulation with signal 1 and signal 2, leading to caspase 1 cleavage without the need for transcriptional upregulation of NLRP3 (Lin et al., 2014). These studies suggested that the effect of LPS does rely on post-translational processes other than transcriptional upregulation of NLRP3. In addition to priming, the second signal, such as potassium efflux induced by the potassium ionophore nigericin or ATP, has also been reported to regulate full activation of the NLRP3 inflammasome at a non-transcriptional level, but the exact mechanism remains elusive. Several post-translational modifications (PTMs), which are thought to represent key molecular switches, have been described to regulate the inflammasome. Classical PTMs such as phosphorylation, ubiquitylation have been studied in the regulation of inflammasome activation (Figure 1). Other less known modifications, such as acetylation (He et al., 2020), ADP-ribosylation (Bose et al., 2014), nitrosylation (Yoon et al., 2015), prenylation (Politiek and Waterham, 2021), citrullination (Ciesielski et al., 2022), and ubiquitin-like conjugation (SUMOylation) (Barry et al., 2018) and Neddylation (Segovia et al., 2015), have recently been reported to contribute to the regulation of inflammasome signaling (Figure 1), but their significance still remains unclear. In this review, we have summarized key PTMs of various inflammasome components and their roles in the regulation of inflammasome activation. Finally, we have highlighted the importance of some of these PTMs in driving various disease pathologies with an emphasis on potential future treatments focused on these particular features.

## 2 Molecular Basis of Inflammasome Assembly

The canonical inflammasomes are composed of three key components, a sensor, in some cases an adaptor, and the effector caspase proteases. There are mainly three classes of sensor molecules which include the NOD-like receptors (NLRs), the AIM2-like receptors (ALRs), and the pyrin sensor. Although the human genome encodes 23 NLRs (Ting et al., 2008), only NLRP1 (Faustin et al., 2007), NLRP3 (Agostini et al., 2004; Mariathasan et al., 2006), NLRP6 (Elinav et al., 2011; Hara et al., 2018), NLRP7 (Khare et al., 2012), NLRP12 (Vladimer et al., 2012), and the NAIP/NLRC4 complex (Miao et al., 2006; Kofoed and Vance, 2011; Ren et al., 2019) have been reported to assemble a functional inflammasome complex. NLRs have tripartite domain organization and consist of: 1) N-terminal CARD or baculovirus inhibitor of apoptosis protein repeat (BIR) or PYD, which mediates homotypic protein–protein interactions for downstream signaling; 2) central nucleotide-binding domain (NBD) or NACHT domain, which elicits ATP-induced oligomerization; and 3) C-terminal manifold series of leucine-rich repeats (LRRs), which are responsible for ligand sensing and autoregulation (Harton et al., 2002; Broz and Dixit, 2016). The NACHT domains of NLRs, despite their structural similarities, are activated by different stimuli, but the underlying reason explaining these differences is unknown.

The structural and molecular basis of NLRP1 activation is unique and different from other NLR family members. The human NLRP1, contains a unique function-to-find domain (FIIND) and a CARD domain at its carboxy-terminus, in addition to an amino-terminal PYD, a NOD, and LRR domains. There is only one human NLRP1, while multiple paralogs of NLRP1 (such as NLRP1a, NLRP1b, and NLRP1c) sharing similar domain organization are found in mice, with the exception that mouse NLRP1 lack PYD. NLRP1 undergoes autolytic proteolysis within the FIIND domain to generate two non-covalently associated fragments that remain in an auto-inhibited state. To explain full activation of NLRP1, a “functional degradation” model has been proposed, which involves either cleavage of the N-terminus of NLRP1 by Anthrax lethal factor or ubiquitylation induced by *Shigella* effector E3-ligase IpaH7.8 resulting in proteasomal degradation of NLRP1, releasing the bioactive C-terminus to bind and activate caspase 1 (Sandstrom et al., 2019). NLRP1 also senses bacteria like *Listeria* (Neiman-Zenevich et al., 2017), intracellular parasite Toxoplasma (Ewald et al., 2014), and viral dsRNA (Bauernfried et al., 2021), but the mechanism of NLRP1 activation by these pathogens or their components has not been elucidated. Moreover, the role of NBD and LRR domains of NLRP1 in the activation of this inflammasome remains to be solved.

AIM2 and IFI16 (interferon gamma-inducible protein) belong to the ALRs family and comprise an N-terminal PYD that interacts with ASC, and a C-terminal HIN (Hematopoietic, interferon-inducible, nuclear localization) domain for the recognition of double-stranded DNA (dsDNA) (Burckstummer et al., 2009; Fernandes-Alnemri et al., 2009; Hornung et al., 2009; Roberts et al., 2009; Rathinam et al., 2010; Kerur et al., 2011). A different type of inflammasome is formed by the sensor pyrin (also known as marenostrin or TRIM20), which contains a PYD domain, a B-box domain (zinc finger), a coiled-coil (CC) domain, and a B30.2/SPRY domain that is absent in murine pyrin) (Heilig and Broz, 2018). Pyrin activates and assembles into the inflammasome complex in response to bacterial toxins that alter homeostasis and cause inactivation of RhoA GTPase (Xu et al., 2014). Activated pyrin interacts with ASC through PYD-PYD homotypic interactions to initiate formation of the inflammasome complex. Upstream sensor proteins such as NLRP3, AIM2, and pyrin require the adaptor protein ASC for inflammasome assembly and activation, and thereby are known as ASC-dependent inflammasomes. Further homotypic interactions of ASC with caspase-1 through homotypic CARD interaction results in inflammasome complex formation (Broz and Dixit, 2016). Other sensors with CARDs (e.g., NLRC4/NAIP and NLRP1) can directly activate caspase-1, and thus represent the ASC-independent inflammasomes. However, reports on the function of the latter show that the presence of ASC enhances IL-1β secretion, although loss of ASC does not affect pyroptosis induced in response to NLRC4 and NLRP1 activators (Broz et al., 2010; Guey et al., 2014).

Classical inflammasome complex formation has not been demonstrated for the non-canonical inflammasomes. Instead, caspase oligomerization and autoactivation are shown to be mediated by direct binding of the CARD domain of caspase-11 with cell wall components of bacteria such as LPS. This binding induces caspase oligomerization and activation, with the subsequent cleavage of gasdermin D (GSDMD) and cell death by pyroptosis (Zanoni et al., 2016).

## 3 Phosphorylation of Inflammasome Components

The most widely studied PTM in the context of inflammasome signaling is protein phosphorylation. In recent years, several comprehensive reviews have been published on this topic and we refer to them for more detailed information (Yang et al., 2017; Liang et al., 2021; McKee et al., 2021; Seok et al., 2021). They often focus on the most studied inflammasome, NLRP3. In this review, we want to recapitulate the main findings in protein phosphorylation of the various inflammasome sensors (NLRs), the adaptor protein ASC (PYCARD) or, effector caspases (Caspase-1 and Caspase-4), and their impact on various inflammasomes.

Kinases carry out the phosphorylation reactions by transferring the gamma phosphate of ATP onto hydroxyl groups of various substrates including lipids, sugars, or amino acids (Fabbro et al., 2015). This process can be reversed by the activity of corresponding phosphatases. The first phosphorylation of proteins was described for casein [by phosphorylase kinase (PHK)] in 1954 (Cohen, 2002a), and since then, over 500 protein kinases have been identified. A schematic of protein phosphorylation process is shown in (Figure 1). High-throughput biochemical and cell-based profiling for protein kinases, in combination with the development of phospho-proteomics has not only allowed identification of substrate protein(s) phosphorylated by their respective kinase(s), but also the specific phosphorylation site(s). The searchable database PhosphositePlus is a collection of putative sites for PTMs, including protein phosphorylation (Hornbeck et al., 2012; Hornbeck et al., 2019). These PTMs have been identified in high-throughput proteomics screens.

Protein kinases of eukaryotes mainly phosphorylate either tyrosine (TPKs; tyrosine-specific protein kinases), serine/threonine (STPKs; Ser-/Thr-specific protein kinases) or both tyrosine and threonine (dual-specificity protein kinases) (Cohen, 2002a, Cohen, 2002b; Cohen et al., 2021; Fabbro et al., 2015). For the main components of inflammasomes—the sensors (NLRs), pyrin, the adaptor protein ASC, and the two inflammasome-associated inflammatory caspases Caspase-1 and Caspase-4, multiple tyrosine, serine, and threonine phosphorylation sites have been identified in recent years. However, relatively few have been functionally characterized to establish the effect of the respective phosphorylation event with regards to inflammasome activity.

The best studied phosphorylation sites will be discussed in this chapter according to their location within or outside the characteristic domains of NLRs, pyrin ASC and the two inflammatory caspases (Table 1). summarizes known phosphorylation sites based on publicly available information obtained from PhosphositePlus and other original research articles. Due to its unique domain structure, pyrin phosphorylation sites are not included in (Table 1), but are as follows: Ser6, Ser11, Thr12, Ser141, Thr177, Ser187, Ser208, Ser209, Ser242, Thr267, Ser273, Ser363, Ser368. Thr672, Ser 673, Tyr 732, and Tyr741.

**TABLE 1:** Summary of human inflammasome phosphorylation. Phosphorylation sites which have been functionally validated are highlighted in green, whereas non-characterized sites found in the PhosphositePlus are depicted in orange (data retrieved from PhosphositePlus, v6.6.0.4 in April 2022).

Protein	Phosphorylation sites and implicated proteins	Inflammasome modulation	Reference
AIM2	Thr254, Thr257, Thr262, Ser322	Unknown	Hornbeck et al. (2012)
NALP1 (NLRP1)	Thr41, Ser291, Ser823, Thr1000	Unknown	Hornbeck et al. (2012)
NLRP2	Ser107, Thr135, Tyr189, Ser320, Ser654, Ser671, Thr834	Unknown	Hornbeck et al. (2012)
NLRP3	Ser5 AKT1, PP2A	Inhibition	Stutz et al. (2017)
	Tyr32 PTEN	Inhibition	Huang et al. (2020)
	Tyr136, Tyr140, Tyr143, Tyr146 all BTK	Activation	Bittner et al. (2021)
	Ser198 JNK1, PAK1	Activation	Song et al. (2017)
	Ser295 PKD	Activation	Zhang et al. (2017)
	Ser295 PKA	Inhibition	Mortimer et al. (2016)
	Thr659 PAK1	Activation	Dufies et al. (2021)
	Ser803 CSNK1A1	Inhibition	Niu et al. (2021)
	Tyr861 PTPN22	Activation	Spalinger et al. (2016)
	Tyr13, Ser161, Ser163, Ser728, Ser894. Ser898, Ser975	Unknown	Hornbeck et al. (2012)
NALP4 (NLRP4)	Ser605	Unknown	Hornbeck et al. (2012)
NLRP5	Ser16, Thr185, Thr191, Ser642, Ser1062, Ser1064	Unknown	Hornbeck et al. (2012)
NLRP6	Tyr72, Ser749	Unknown	Hornbeck et al. (2012)
NLRP7	Thr2, Ser3, Thr61, Thr81, Ser285, Tyr318, Thr732, Thr734, Thr737, Tyr810, Thr849	Unknown	Hornbeck et al. (2012)
NLRP8	Ser544, Ser935	Unknown	Hornbeck et al. (2012)
NLRP9	Tyr139, Tyr140, Ser269, Ser270, Ser691, Tyr693, Ser952	Unknown	Hornbeck et al. (2012)
NALP10 (NLRP10)	Thr36, Ser38, Ser268, Tyr323, Tyr327, Thr377, Thr511, Ser639, Thr640	Unknown	Hornbeck et al. (2012)
NLRP11	Ser439, Tyr446, Ser940, Ser943	Unknown	Hornbeck et al. (2012)
NLRP12	Ser192, Ser224, Ser727, Ser979	Unknown	Hornbeck et al. (2012)
NLRP13	Thr243, Thr358	Unknown	Hornbeck et al. (2012)
NLRP14	Ser302, Thr310, Thr311, Ser312, Tyr443, Thr1022, Ser1040	Unknown	Hornbeck et al. (2012)
NLRC4	Ser533 PRKCD, LRRK2	Activation	Qu et al. (2012)
	Thr736	Unknown	Liu et al. (2017a)
			Hornbeck et al. (2012)
Caspase-1	Ser376 PAK1	Activation	Basak et al. (2005)
	Ser16, Thr21, Thr32, Ser149, Thr226, Ser227, Ser306	Unknown	Hornbeck et al. (2012)
Caspase-4	Ser16, Ser83, Ser271, Ser274, Thr352	Unknown	Hornbeck et al. (2012)
PYCARD (Asc)	Thr16 IKKα	Inhibition	Martin et al. (2014)
	Tyr60	Activation	Mambwe et al. (2019)
	Tyr137	Activation	Chung et al. (2016)
	Tyr146 Pyk2, cAbl	Activation	Gavrilin et al. (2021)
	Ser195 IKKα	Inhibition	Martin et al. (2014)
	Ser46, Ser164	Unknown	Hornbeck et al. (2012)

### 3.1 Pyrin Domain Phosphorylation

The pyrin domain (PYD) is found in the N-terminus of inflammasome sensors and the adaptor protein ASC, enables protein-protein interaction between the inflammasome sensor and the adaptor protein, a critical step in inflammasome activation. So far, functionally characterized phosphorylation sites in this domain for NLRP3 have generally been demonstrated to have an inhibitory effect on inflammasome activation.

Ser5 is located in helix 1 of the human NLRP3 PYD at a protein-protein interaction surface with ASC, inhibits the NLRP3 inflammasome in macrophages. Furthermore, the authors have demonstrated that the two negative charges introduced by phospho-serine were able to disrupt the PYD-PYD interaction between NLRP3 and ASC and thereby prevents the activation of this inflammasome (Stutz et al., 2017). The upstream kinase catalyzing Ser5 phosphorylation is AKT (protein kinase B), and pharmacological inhibition of this enzyme resulted in increased NLRP3 inflammasome activity *in vitro* and *in vivo* (Zhao et al., 2020). Interestingly, the same researchers also discovered a crosstalk between protein phosphorylation and ubiquitination. Ser5 phosphorylation stabilizes NLRP3 by reducing its ubiquitination on Lys496, which inhibits its proteasome-mediated degradation by the E3-ligase Trim31. Dephosphorylation of Ser5 by Protein Phosphatase 2 A (PP2A) is essential for NLRP3 inflammasome activation as demonstrated by a drastically reduced NLRP3 activation when PP2A was inhibited or down-regulated (Stutz et al., 2017). An additional layer of fine-tuning NLRP3 inflammasome activation is mediated by Bruton’s tyrosine kinase (BTK) (Mao et al., 2020). BTK is bound to NLRP3 during the priming phase of inflammasome activation and inhibits PP2A-mediated dephosphorylation of Ser5 in the PYD domain of NLRP3, consequently BTK inhibitors have been shown to suppress NLRP3 inflammasome activation and IL-1β secretion (Ito et al., 2015).

Another phosphorylation site in the PYD domain of NLRP3 involved in controlling its protein-protein interaction with ASC is Tyrosine 32. While the upstream kinase is currently unknown, the lipid and protein phosphatase PTEN can directly dephosphorylate Tyr32 (Huang et al., 2020), and in turn facilitated NLRP3-ASC interaction, inflammasome assembly and activation.

The interaction between a NLR and ASC by protein phosphorylation can not only be controlled through phosphorylation and dephosphorylation of the inflammasome sensor, but also at the level of the inflammasome adaptor protein. There are two putative phosphorylation sites located in the PYD domain of ASC: Ser46 and Tyr60. The latter was identified and functionally characterized by Mambwe and colleagues by using the pharmacological protein tyrosine phosphatase (PTPase) inhibitor phenylarsine oxide (PAO), which prevented AIM2 and NLRP3 inflammasome activation in human and mouse macrophages. Moreover, site-directed mutagenesis of ASC tyrosine residue Tyr60 to phenylalanine, a non-phosphorylatable tyrosine mimic led to a significant reduction in inflammasome activity measured by IL-1β release (Mambwe et al., 2019), highlighting the role of Tyr60 phosphorylation for inflammasome assembly and function. However, it remains unclear if Tyr60 phosphorylation has a direct effect on AIM2/NLRP3 protein-protein interaction with ASC.

Subcellular location of ASC is dependent on its phosphorylation status. The protein kinase IKKα phosphorylates murine ASC in the PYD domain at serine 16 (Thr16 in human) and at Ser193 within the CARD domain (human Ser195) (Martin et al., 2014). Phosphorylation at these two sites is important for the nuclear localization of an ASC/IKKα protein complex. Signal 1 of the inflammasome activation leads to PP2A-dependent dephosphorylation of ASC and subsequent release of ASC into the cytosol for inflammasome assembly. Loss of IKKα kinase activity results in markedly enhanced NLRP3, AIM2 and NLRC4 inflammasome activation, with aberrant caspase-1 activation and IL-1β secretion (Martin et al., 2014).

Given this evidence that phosphorylation events in this domain negatively impact the interaction between the inflammasome sensor and ASC, one can speculate that phosphorylation events for other NLRPs might have negative effects on their inflammasome activation. Table 1 summarizes the putative phosphorylation site in this domain beyond the NLRP3 and ASC. It would be interesting to experimentally test role of these putative phosphorylation sites in other NLRs on their respective inflammasome activation. Due to its different molecular mechanism of activation, an exception from this hypothesis could be NLRP1 (Mitchell et al., 2019; Sandstrom et al., 2019). NLRP1 is kept in an autoinhibited confirmation and activation requires autoproteolysis, a process termed “functional degradation” (Sandstrom et al., 2019). It would be interesting to see, if the putative phosphorylation site at Thr41 in the PYD or for that matter other phosphorylated amino acid residues could lead to an inflammasome activation. The upstream kinase facilitating this phosphorylation is currently unknown, but it could provide an alternative molecular mechanism to activate the NLRP1 inflammasome.

### 3.2 NACHT Domain Phosphorylation

NLR proteins contain a central NACHT domain, which possess nucleoside-triphosphatase (NTPase) activity. In a recent review, Sandall and colleagues highlight the importance of ATP binding and hydrolysis for inflammasome activation (Sandall et al., 2020).

The NLRP3 ATPase activity is sensitive to phosphorylation at Ser295 by protein kinase PKA, as demonstrated by Mortimer et al. (2016). Interestingly, phosphorylation at Ser295 does not only hinders the ATPase activity, but also induces protein ubiquitylation, which inhibited the inflammasome independently of protein degradation (Guo et al., 2016). However, Zhang et al. (2017) report an opposing effect of NLRP3 Ser295 phosphorylation when phosphorylated by the protein kinase PKD. Under these experimental conditions, the phosphorylation of Ser295 is important for NLRP3 inflammasome activation. Further investigation is needed to fully understand the opposing effects of NLRP3 Ser295 phosphorylation by PKA and PKD. Spatial and temporal effects of protein phosphorylation could offer a possible explanation for the opposite effects of Ser295 phosphorylation. PKD mediated phosphorylation of Ser295 occurs when NLRP3 is localized at mitochondria-associated membranes (MAM) and is required for the release of NLRP3, the subsequent cytosolic localization, ASC recruitment and inflammasome complex formation (Zhang et al., 2017). PKA-mediated phosphorylation occurs in the cytosol and prevents conformational changes crucial for inflammasome complex formation (Mortimer et al., 2016).

Despite the identification of other putative phosphorylation sites in various inflammasome sensor proteins (Table 1), little is known what these events do to the activation status of the respective inflammasome. Given the inhibitory effect of Ser295 phosphorylation for NLRP3 inflammasome activity by interfering with ATPase-dependent structural changes, one can speculate that phosphorylation in the proximity of ATP binding leads to inhibition of inflammasome activity. A distinct motif in the NACHT domain of NLR proteins is the Walker-A “P-loop” motif with the consensus sequence of GxxGxGK [T/S]. The functionally important Walker-A motif binds the phosphate groups of ATP and catalyzes phosphoryl transfer (Kawabori et al., 1978). Phosphorylation of the threonine or serine residue within the Walker-A motif has been detected for two NLR proteins: NLRP3 (Thr233) and NLRP12 (Ser224) (Hornbeck et al., 2012; Hornbeck et al., 2019). Upstream kinases responsible for these two phosphorylation events are yet to be identified but could provide an alternative to modulate NLRP3 or NLRP12 activities.

### 3.3 Leucine-Rich Repeat Domain Phosphorylation

NLRs (apart from NLRP10) contain leucine-rich repeat (LRR) domain, usually located towards the C-terminus. The role of this domain includes auto-inhibition and ligand sensing. Several phosphorylation sites were identified in various LRR domains and can be found summarized in (Table 1).

The protein-protein interaction between NLRP3 and NEK7, a member of the family of mammalian NIMA-related kinases (NEK proteins) has been identified to be important for inflammasome assembly and activation (He et al., 2016; Schmid-Burgk et al., 2016; Shi et al., 2016; Sharif et al., 2019). NLRP3-activating stimuli promoted the NLRP3-NEK7 interaction in a process that is dependent on potassium efflux (He et al., 2016), but the kinase activity of NEK7 is dispensable and just the protein-protein interaction between NLRP3 and the catalytic domain of NEK7 is sufficient for the activation of the NLRP3 inflammasome. NEK7 plays a scaffolding role in the activation of the NLRP3 inflammasome complex by bridging adjacent NLRP3 subunits with bipartite interactions (Sharif et al., 2019). Recently, the phosphorylation of Ser803 (situated in the third LRR domain) in NLRP3 has been demonstrated to inhibit NEK7-NLRP3 interaction (Niu et al., 2021). Phosphomimic substitutions of Ser803 abolish NEK7 recruitment and inflammasome activity in macrophages *in vitro* and *in vivo*. Full-length, membrane-associated NLRP3 possess a double-ring cage structure, which is facilitated by LRR-LRR and PYD-PYD interactions (Andreeva et al., 2021). Ser803 is located close to positively charged residues from the adjacent LRR (e.g., R1009), and the phosphorylation likely stabilizes the NLRP3 cage structure. The kinase facilitating this PTM has been identified as CSNK1A1, while the phosphatase that could counteract this phosphorylation remains elusive.

Phosphorylation at Ser891/895 in mouse macrophages (corresponding to Ser894/898 in human) enables the binding of the E3-ligase TrCP1 in (Ubiquitination chapter), which in turns catalyzes the proteasomal degradation of NLRP3 *via* ubiquitination at Lys380 (Wang D. et al., 2021). While the upstream kinase remains to be identified, the NLRP3 can be stabilized by the Hippo pathway protein YAP, which blocks the TrCP1/NLRP3 interaction.

NLRP3 has been shown to be phosphorylated at Tyr861 by an unknown kinase, allowing its interaction with the autophagy machinery Sequestosome 1 (SQSTM1) to target to the phagophore, and thereby control inflammasome activation (Spalinger et al., 2016). Protein tyrosine phosphatase non-receptor 22 (PTPN22) counteracts this mechanism in an ASC-dependent manner to dephosphorylates pTyr861, allowing NLRP3 inflammasome assembly (Spalinger et al., 2017).

### 3.4 Hematopoietic, Interferon-Inducible, Nuclear Localization-200 Domain

The C-terminal HIN-200 domain binds to dsDNA through electrostatic attraction between the positively charged HIN domain residues and the dsDNA sugar-phosphate backbone (Jin et al., 2012). AIM2 is kept in an autoinhibited confirmation through PYD and HIN-200 domain protein-protein interaction, which is liberated by dsDNA binding (Jin et al., 2012). The PYD domain is then released to engage with the downstream inflammasome adaptor protein ASC through homotypic PYD-PYD interactions.

Three phosphorylation sites in the HIN-200 domain of AIM2 have been identified (Table 1): Thr254, Thr257, and Thr262 (Hornbeck et al., 2012; Hornbeck et al., 2019). One could speculate that the negative charges introduced by phosphorylation could interfere with the dsDNA binding to the HIN-200 domain of AIM2. If this hypothesis is true, the upstream kinase responsible for these phosphorylation events, once identified, could provide an attractive therapeutic target for diseases of aberrant AIM2 activation from self-dsDNA, a key driver of inflammatory and autoimmune diseases (Man et al., 2016).

### 3.5 CARD Domain Phosphorylation

The CARD domain, through protein-protein interactions, enables the formation of inflammasome complexes and unsurprisingly, protein phosphorylation has been identified as a mechanism controlling this process. Two phosphorylated tyrosine residues (Tyr137 and Tyr146) in ASC have been functionally characterized. When Mambwe and colleagues established that the pharmacological PTPase inhibitor PAO had the profound inhibitory effect on the AIM2 and NLRP3 inflammasomes respectively, in human and murine macrophages (Mambwe et al., 2019). They identified the CARD domain-located tyrosine Tyr137 to be important for AIM2 and NLRP3 inflammasomes assembly and function. In their study, the authors built on previous studies, which identified Tyr146 phosphorylation as crucial signal controlling ASC function (Hara et al., 2013; Chung et al., 2016; Darweesh et al., 2019; Gavrilin et al., 2021). Several kinases have been associated with directly or indirectly controlling the phosphorylation status: Syk, Jnk (Hara et al., 2013), Pyk2 (Chung et al., 2016), protein kinase R (PKR) (Darweesh et al., 2019) and cAbl (Gavrilin et al., 2021). This demonstrates that ASC regulation through tyrosine phosphorylation is a very multifaceted system, which might involve many cells type stimulation- and kinase redundancy-dependent layers of complexity.

Very little is known how Caspase activity is regulated by CARD domain phosphorylation despite the identification of several putative phosphorylation sites (Table 1). One could speculate that Caspase-1 or Caspase-4 protein-protein interaction with ASC could be affected by phosphorylation in the respective CARD domains, however this needs to be experimentally tested.

### 3.6 Peptidase Domain Phosphorylation

Caspase-1 and Caspase-4 are directly inflammasome-associated proteases in human cells, whereas Caspase-11, is the Caspase-4 orthologue in mice. All three caspases have in common that the proteolytic cleavage of their respective substrates is catalyzed by their peptidase domains. Several phosphorylation sites have been found in this domain (Table 1), but so far only one has been functionally characterized.

Caspase-1 is phosphorylated by the protein kinase p21-activated kinase 1 (PAK1) at Ser376 upon *Helicobacter pylori* LPS treatment of THP-1 cells (Basak et al., 2005). The LPS-induced Caspase-1 activation was abrogated in cells transfected with Caspase-1 (Ser376Ala) mutant. This demonstrates that this PTM is important for Caspase-1 activation and given its conservation as Thr352 in Caspase-4, it would be interesting to study if Caspase-4 activity upon inflammasome stimulation could be regulated via a similar phosphorylation-dependent mechanism.

### 3.7 Inter-domain Phosphorylation

Many phosphorylation sites are situated in between the distinct protein domains of NLRs (see Table 1). The NLRP3 inflammasome has been studied extensively and some of the interdomain phosphorylation sites have been functionally characterized.

BTK kinase activity inhibits PP2A function and thereby lead to inhibition of NLRP3 de-phosphorylation of Ser5 of NLRP3 in PYD domain preventing its oligomerization (Mao et al., 2020). Recently, four tyrosine residues have been identified by Bittner et al. (2021) as direct BTK phosphorylation upon *in vitro* and *in vivo* inflammasome activation. These sites are Tyr136, Tyr140, Tyr143, and Tyr164, that are situated in the polybasic linker between PYD and NACHT domains, which is critical for interaction with negatively charged phosphatidylinositol phosphates (PIPs) at organelles, such as the Golgi. Ablation of BTK kinase activity or mutation of the respective tyrosine residues decreased the formation of cytosolic NLRP3 oligomers, complexes with ASC and IL-1β release (Bittner et al., 2021). This study confirms previous studies demonstrating a physical interaction of BTK with NLRP3 and ASC as well as BTK inhibition by kinase inhibitors or in knock-out mice blocks NLRP3 inflammasome activation (Ito et al., 2015; Liu X. et al., 2017). The BTK-mediated tyrosine phosphorylation at the above-mentioned residues is introducing negative charges, which could weaken the interaction between NLRP3 and phospholipids at the Golgi and the relevance of such a disruption has been proven previously (Chen and Chen, 2018). The contradicting effects of BTK kinase-mediated phosphorylation events (Ser5 vs. Tyr136, Tyr140, Tyr143, and Tyr164) for NLRP3 inflammasome activity highlight again the temporal and spatial control of the NLRP3 inflammasome through protein phosphorylation/dephosphorylation.

Phosphorylation of mouse NLRP3 at Ser198 (mouse Ser194) is catalyzed by c-Jun N-terminal kinase 1 (JNK1) in response to chronic LPS treatment or other TLR ligands (Song et al., 2017), in a transcription-independent manner. This phosphorylation even is crucial for inflammasome activation by promoting NLRP3 self-association. In addition, phosphorylation of Ser198 enables inflammasome activation by promoting NLRP3 deubiquitylation by BRCC3, a component of the cytosolic BRISC complex (Py et al., 2013). This crosstalk between phosphorylation and ubiquitylation was further proven by Ren and colleagues, who identified ABRO1, a subunit of the BRISC deubiquitylate complex, to be necessary for optimal NLRP3-ASC complex formation, ASC oligomerization, caspase-1 activation, and IL-1β and IL-18 production upon treatment with NLRP3 ligands after the priming step, indicating that efficient NLRP3 activation requires ABRO1 (Ren et al., 2019).

As discussed for Ser803 phosphorylation (LRR domain), the interaction between NEK7 and NLRP3 is crucial for its inflammasome activation, the NLRP3-NEK7 binding induces NLRP3 deubiquitylation by BRCC3 and subsequently inflammasome assembly, with NLRP3 phosphomimetic mutants showing enhanced ubiquitination and degradation than wildtype NLRP3 (He et al., 2016; Niu et al., 2021). While Ser803 phosphorylation is catalyzed by the protein kinase CSNK1A1, recently Dufies and colleagues identified Thr659 as an important site for NLRP3 and NEK7 protein-protein interaction (Dufies et al., 2021). In an *Escherichia coli* infection model system, PAK1 phosphorylates Thr659, which in turn facilitates NLRP3-NEK7 interactions, inflammasome formation and IL-1β cytokine maturation. PAK1 appears to be key regulator in the anti-bacterial host-responses by phosphorylating Thr659 on NLRP3 and Ser376 of Caspase-1.

A key inflammasome sensors responsible for the immune response to bacterial infections is the NLRC4 inflammasome (Amer et al., 2006; Franchi et al., 2006; Miao et al., 2006). NLRC4 is phosphorylated at Ser533 phosphorylation (Qu et al., 2012), and mutation of serine 533 to alanine (Ser533Ala) did not activate Caspase-1 and pyroptosis in response to *Salmonella typhimurium*, indicating that S533 phosphorylation is critical for NLRC4 inflammasome function. Conversely, phosphomimetic NLRC4 S533D caused rapid macrophage pyroptosis without infection. The phosphorylation site Ser533 is conserved among the NLRC4 proteins from different species, which suggests that it is potentially important for the structure or function of NLRC4. A screen of kinase inhibitors identified PRKCD (PKCδ) as a candidate kinase upstream of Ser533 phosphorylation (Hornbeck et al., 2019) and, PKCδ phosphorylated NLRC4 S533 *in vitro*, whilst immunodepletion of PKCδ from macrophage lysates blocked NLRC4 S533 phosphorylation *in vitro* (Hornbeck et al., 2019). However, more recent studies discovered a more complex NLRC4 biology *in vivo*. NLRC4 Ser533Ala knock-in mice were generated by two groups independently and used in *S. Tryphimurium in vivo* infection models and in *ex vivo* mechanistic studies using bone marrow derived macrophages (BMDMs). Both studies demonstrated that the phosphorylation at Ser533 had no to very subtle effect for NLRC4 inflammasome activation and the response to *S*. Tryphimurium infection. While Qu and colleagues observed a delayed, but not completely blocked response and a potential crosstalk with the NLRP3 inflammasome (Qu et al., 2012), however, by using a Nlrc4-deficient mouse line, mice with Ser533Asp phosphomimic or Ser533Ala non-phosphorylatable NLRC4, Tenthorey and colleagues did not observe a requirement for Ser533 phosphorylation in NLRC4 inflammasome function nor any involvement of NLRP3 inflammasome (Tenthorey et al., 2020). A possible explanation for the divergent results could be different experimental conditions, such as animal housing, different cell culture conditions for BMDMs.

A second kinase with the ability to phosphorylate NLRC4 at Ser533 is LRRK2 (Liu W. et al., 2017). LRRK2 has been found to form a complex with NLRC4 in the macrophages, and the formation of the LRRK2-NLRC4 complex led to the phosphorylation of NLRC4 at Ser533 (Liu W. et al., 2017). Importantly, the kinase activity of LRRK2 is required for optimal NLRC4 inflammasome activation, whereas LRRK2 deficiency reduced Caspase-1 activation and IL-1β secretion in response to NLRC4 inflammasome activators in macrophages (Liu W. et al., 2017). Future studies are required to investigate the temporal and spatial control of NLRC4 Ser533 phosphorylation and redundancies/synergies between the two kinases PKCδ and LRRK2 *in vitro* and *in vivo* models.

**FIGURE 2:**
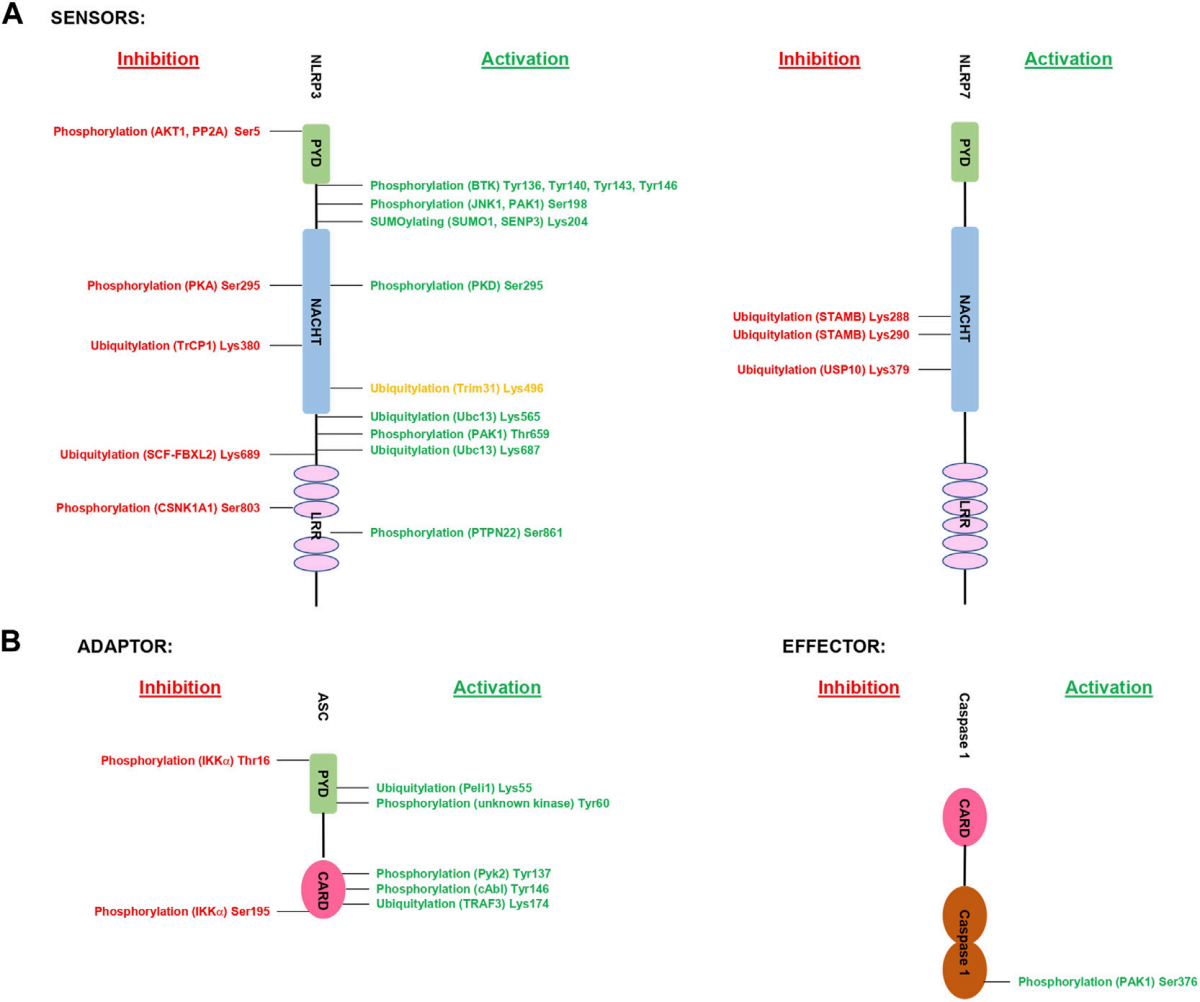
Summary of validated PTMs of inflammasome components. The figure shows the validated PTMs of inflammasome components and the responsible proteins of each modification. Highlighted in green are validated PTMs with an increased inflammasome activity, while PTMs in red indicate validated sites. The site in orange requires further validation of its downstream functional effect. 2 A shows the sensors and 2B the downstream molecules.

Taken together, phosphorylation of inflammasome components occur at various stages of activation or inhibition of these multimeric protein complexes. From controlling the priming step (e.g., NLRP3 Ser198), to inflammasome complex formation (e.g., NLRP3 Ser803), the subcellular localization (e.g., NLRP3 Tyr136, Tyr140, Tyr143 and Tyr164) and to protein stability (e.g., NLRP3 Ser5). The validated residues that are phosphorylated in inflammasome components so far are represented in (Figures 2A,B). Future studies are needed to provide a deeper understand of:

1) upstream kinases and corresponding protein phosphatases
2) redundancies between kinases for phosphorylation events
3) temporal and spatial control of phosphorylation events
4) functional characterization of identified phosphorylation sites, in particular beyond the NLRP3 inflammasome.

## 4 Ubiquitylation of Inflammasome Components

From its discovery in the late 1970’s and early 1980’s by Aaron Ciechanover, Avram Hershko, and Irwin A, the ubiquitin system has gained importance, and this PTM is currently investigated intensely, and it is crucial in regulating several biological processes (Hershko et al., 1979; Komander, 2009). In recent years several ubiquitin related proteins and modifications have been linked to inflammasome components. The ubiquitin system is mainly formed by 3 coupled enzymes where E1-E2-E3 can add ubiquitin molecule(s) to a substrate protein. E1 or ubiquitin activating enzyme forms a thioester bond with a ubiquitin in a ATP-dependent manner, in a second step the E2 ubiquitin-conjugating enzyme forms a ubiquitin-thioester intermediate that is finally transferred to the substrate protein by an E3-ligase. The addition of a single ubiquitin molecule to a protein leads to generation of a mono-ubiquitylated species, but a protein is referred to as multi mono-ubiquitylated if several ubiquitin molecules attached to the same protein at different position. Two or more ubiquitin molecules can be attached to each other to generate poly-ubiquitin chains, and depending on Lys residue used, seven different types of poly-ubiquitin chains can be generated. Moreover, a distinct type of ubiquitin chain called M1 or linear poly-ubiquitin chains is formed when ubiquitin molecules are arranged in a head-to-tail fashion via the C-terminal carboxy group of the donor ubiquitin and N-terminal methionine of the acceptor ubiquitin (Figure 1). In the physiological signaling processes, mixed and branched ubiquitin chains have been shown to play a vital role in bringing protein complexes to the close proximity of each other for downstream signaling cascade (Emmerich and Cohen, 2015; Akutsu et al., 2016; Cohen and Strickson, 2017). Additionally, this process is regulated by a reversible process mediated by deubiquitylating enzymes (DUBs).

The importance of ubiquitylation in the regulation of the inflammasome activation has been well recognized, and several comprehensive reviews on this topic have been published (Yang et al., 2017; Lopez-Castejon, 2020), but since then, many more have been described indicating the importance of this PTM in the regulation of inflammasome activation. A more recent review by Kessler and colleagues (Liang et al., 2021) brings attention to this process, but with a major focus on the most studied inflammasome sensor NLRP3. Here we have summarized ubiquitylation regulating NLRP3 inflammasome and discussed ubiquitylation of other sensors, the adaptor protein ASC and effector proteins Caspase-1 and Caspase-4. Ubiquitylation has been linked to the inflammasomes, but compared to the protein phosphorylation, it has been studied less, and therefore fewer ubiquitylated sites residues have been found and validated so far. This makes it difficult to summarize what is known to date by proteins’ domain structure. Therefore, regarding this PTM we will summarize the different inflammasome components one by one.

### 4.1 NOD-Like Receptors

#### 4.1.1 NLRP1

NLRP1 was the first inflammasome described (Martinon et al., 2002), although its biology and function remained largely elusive, until recent publications discovered that the Lethal Factor (LF) action of the anthrax toxin is able to activate by inducing N-terminal degradation of NLRP1b by the N-end rule proteasome pathway (Chui et al., 2019). The E3-ligase IpaH7.8, a *Shigella* flexneri ubiquitin ligase secreted effector, can induce NLRP1b activation and degradation by ubiquitylation (Sandstrom et al., 2019). More recently it has been demonstrated that TRIM25 is able to ubiquitylate human NLRP1 although the type of ubiquitin chain modification and the ubiquitylation site have not been identified so far (Wang X. et al., 2021). Again, in PhosphositePlus can be found several lysines of NLRP1 as potential ubiquitylation sites including Lys297, Lys340, Lys826, and Lys855 identified by massive screenings, but they have not yet been demonstrated experimentally (Hornbeck et al., 2012; Hornbeck et al., 2019).

#### 4.1.2 NLRP3

NLRP3 ubiquitylation has been summarized very well in the reviews by Akther et al. 2021, Moretti and Blander. (2021) and Zangiabadi and Abdul-Sater. (2022). NLRP3 is the receptor where more residues have been identified and validated experimentally. It is curious that only one of the validated Lys residues (Lys496) is recorded in the PhosphositePlus website (Tang et al., 2020; Zhao et al., 2020) (Table 2).

**TABLE 2:** Summary of human inflammasome ubiquitylation. In orange are represented residues non validated by recorded in PhosphositePlus whereas in green are validated residues.

Protein	Ubiquitin sites and implicated proteins	Inflammasome modulation	Reference
AIM2	Lys38, Lys235	Unknown	Akimov et al. (2018)
NALP1 (NLRP1)	Lys297, Lys340, Lys826, Lys855	Unknown	Akimov et al. (2018)
NLRP2	Lys128, Lys567, Lys736, Lys1037	Unknown	Akimov et al. (2018)
NLRP3	Lys496 Trim31	Stabilization	Zhao et al. (2020)
	Lys380 TrCP1	Degradation	Wang et al. (2021a)
	Lys565 Ubc13	Activation	Ni et al. (2021)
	Lys687 Ubc13	Activation	Ni et al. (2021)
	Lys689 SCF-FBXL2	Degradation	Han et al. (2015)
NALP4 (NLRP4)	Lys601	Unknown	Akimov et al. (2018)
NLRP5			
NLRP6			
NLRP7	Lys532, Lys683, Lys817	Unknown	Akimov et al. (2018)
	Lys288 STAMBP	Inhibition	Bednash et al. (2017)
	Lys290 STAMBP	Inhibition	Bednash et al. (2017)
	Lys379 USP10	Stability	Li et al. (2021a)
NLRP8			
NLRP9			
NALP10 (NLRP10)			
NLRP11	Lys264, Lys286, Lys304, Lys655, Lys663, Lys683, Lys750, Lys871, Lys917	Unknown	Akimov et al. (2018)
NLRP12			
LRP13			
NLRP14	Lys1033, LYS1036	Unknown	
NLRC4			
Caspase-1	Lys37, Lys44, Lys53, Lys134, Lys148, Lys158, Lys204, Lys268, Lys274, Lys278, Lys319, Lys320, Lys325	Unknown	Akimov et al. (2018)
Caspase-4	Lys87, Lys107, Lys129	Unknown	Akimov et al. (2018)
PYCARD (Asc)	Lys21, Lys22, Lys109, Llys139	Unknown	Akimov et al. (2018)
	Lys55 Peli1	Activation	Zhang et al. (2021)
	Lys174 TRAF3	Activation	Guan et al. (2015)
GSDMD	Lys43, Lys51, Lys55, Lys62, Lys145, Lys168	Unknown	Akimov et al. (2018)
	Lys236, Lys248, Lys299	Activation	Wagner et al. (2011)
	Lys203 SYVN1	Activation	Shi et al. (2022)
	Lys204 SYVN1		Shi et al. (2022)
IL-1β	Lys133	Degradation	Vijayaraj et al. (2021)

Several DUBs have been linked to the deubiquitylation of NLRP3. DUB that acts a negative regulator of NLRP3 is STAMBP, which removes Lys63 poly-ubiquitin chains from NLRP3 (Bednash et al., 2021).

Other DUBS have been linked to NLRP3 activation. In a study Juliana et al. (2012) demonstrated that TLR4 through MyD88 can activate NLRP3 by inducing its deubiquitylation. They demonstrated that this process is inhibited by antioxidants and is dependent on mitochondrial reactive oxygen species production. In different studies, BRCC3 DUB has been shown to mediate the deubiquitylation of NLRP3 in its LRR Domain (Py et al., 2013; Rao et al., 2019). Moreover, a recent report described that R779C, a gain of function mutation of NLRP3, promotes NLRP3 de-ubiquitylation by BRCC3 and JOSD2 to promote NLRP3 activation, which increases the risk of developing Very-early-onset inflammatory bowel disease (VEOIBD), a chronic inflammatory disease of the gastrointestinal tract can happen in early childhood (Zhou et al., 2021).

Palazon-Riquelme et al. (2018) demonstrated that USP7 and USP47 are DUBs that are able to mediate NLRP3 activation, which is independent of the ability of these DUBs to remove Lys48 or Lys63 poly-ubiquitin chains. UAF1/USP1 complex activates NLRP3 after deubiquitylation through Lys48 poly-ubiquitin chains and thus increases the level of NLRP3 intracellular levels by protecting it from degradation (Song et al., 2020). UCHL5 deubiquitylation activity has been linked to NLRP3 inflammasome activation in the context of Chronic hepatitis C virus (HCV) infection but the ubiquitin chain types or ubiquitylation sites are unknown (Ramachandran et al., 2021). ABRO1, a subunit of BRISC deubiquitylase complex, binds to NLRP3 in a phosphorylation-dependent manner through Ser194 to mediates the cleavage of Lys63 poly-ubiquitylation of NLRP3 leading to its activation (Ren et al., 2019). More recently, it has been demonstrated that USP30 activates the NLRP3 inflammasome by its deubiquitylation. Inhibition of USP30 *via* MF-094, a potent selective inhibitor of this DUB, resulted in decreased protein levels of NLRP3 leading to decreased NLRP3 protein level and consequent suppression of caspase 1 activation (Li et al., 2022).

Several E3-ligases have been shown to regulate NLRP3 inflammasome activity; in some cases, they trigger NLRP3 activation, but majority of them act as suppressor of NLRP3 inflammasome assembly.

#### E3-ligases that deactivate NLRP3 inflammasome formation

A recent paper described E3-ligase SYVN1 as a negative regulator of NLRP3 inflammasome, which causes proteasomal degradation of BRCC3 by attaching Ly48 poly-ubiquitin chains. Since BRCC3 is a DUB that mediates Lys48 deubiquitylation of NLRP3 (Py et al., 2013; Rao et al., 2019) degradation of BRCC3 induce NLRP3 downregulation (Zhang et al., 2022). Another E3-ligase that has been identified as a suppressor of NLRP3 inflammasome is Speckle-type BTB-POZ protein (Spop). Spop directly interacts with NLRP3 and promotes NLRP3 degradation *via* its Lys48 poly-ubiquitination (Wang et al., 2022). On the other hand, β-TrCP1 E3-ligase acts as a brake of the NLRP3 inflammasome formation by attaching Lys27 poly-ubiquitin chains at Lys380 interaction of NLRP3 leading to its degradation *via* proteasomal degradation, although this process can be impaired by the transcription coactivator YAP, which blocks the TrCP1-NLRP3 interaction (Wang D. et al., 2021).

A recent report has shown that the E3-ligase Tripartite motif-containing protein 65 (TRIM65) inhibits NLRP3 activation. The authors demonstrated that TRIM65 binds to the NACHT domain of NLRP3, promotes Ly48 and Lys63 ubiquitination of NLRP3 to prevent NEK7-NLRP3 interaction, and therefore inhibits NLRP3 inflammasome activation (Tang et al., 2021). Other members of this family, including the E3-ligase TRIM24 has been shown to interact with NLRP3, and mediate its ubiquitination although no ubiquitin chains types have been identified in the context of endometriosis context, a disease liked to inflammation that curses with abnormal growth of endometrial tissues outside the endometrium and myometrium of the uterus (Hang et al., 2021). In IgA nephropathy, one of the most common primary glomerulonephritis, has been linked to aberrant activation of NLRP3 inflammasome, TRIM40 has been shown to suppress NLRP3 activation by inducing its ubiquitylation and degradation, consequently loss of TRIM40 leads to overactivation of NLRP3 and proliferation of glomerular mesangial cells (Shen et al., 2021). Another negative regulator, TRIM31 directly binds to the PYD domain of NLRP3 *via* its N-terminal RING domain and induces Lys48-linked poly-ubiquitination and degradation of NLRP3 (Song et al., 2016), and therefore, supresses NLRP3 inflammasome activation. Moreover, as described in the phosphorylation section, Zhao et al. (2020) described that Ser5 phosphorylation stabilizes NLRP3 by reducing its ubiquitination on Lys496, which inhibits its proteasome-mediated degradation by the E3-ligase TRIM31.

NLRP3 has been reported to be Lys48 polyubiquitylated leading to its proteasomal degradation by March 7 E3-ligase, a negative regulator (Yan et al., 2015). More recently, it has been demonstrated that USP5 DUB (ubiquitin specific peptidase 5) plays an important role in restraining the activation of the NLRP3 inflammasome independent of its deubiquitinating activity. Instead, USP5 recruits the E3-ligase MARCHF7/MARCH7 which promotes Lys48 poly-ubiquitin chains in NLRP3 and mediates its degradation through the autophagy-lysosomal pathway (Cai et al., 2021). Another E3-ligase, SCF-FBXL2, has been described to negatively regulate NLRP3 inflammasome activation. The authors demonstrated that NLRP3 is ubiquitylated by SCF-FBXL2 at Lys689 to promote NLRP3 degradation *via* proteasome. Indirectly, in this work, they also demonstrated that another E3-ligase FBXO3-mediated ubiquitination and degradation of SCF-FBXL2, which increases the NLRP3 levels and activity (Han et al., 2015). ARIH2, an E3-ligase called Ariadne homolog 2, interacts with NLRP3 *via* the NACHT domain to mediate NLRP3 ubiquitylation through Lys48 and Lys63 polyubiquitin chains to impairs inflammasome assembly and activation (Kawashima et al., 2017). Moreover, it has been reported that Cullin1, an E3-ligase, and part of the Skp1-Cullin-1-F-boxE3 ligase complex interacts also with NLRP3 by its PYD domain and promotes its Lys63 poly-ubiquitylation and inhibits inflammasome activation (Wan et al., 2019).

The mechanism by which Casitas-B-lineage lymphoma protein-b (Cbl-b), a RING-finger E3 ubiquitin ligase, inhibits NLRP3 activation, is intricate. Cbl-b is recruited via its interaction with Lys63 chains that are synthesized by RNF125 E3-ligase that are attached to the NLRP3 protein. Cbl-b then mediates Lys48 poly-ubiquitylation of NLRP3 at Lys496 in the NBD domain and induces its proteasomal degradation (Tang et al., 2020). It is less evident how the E3-ligase Parkin inhibits NLRP3 inflammasome activation since no ubiquitin chain types or specific sites have been reported so far (Mouton-Liger et al., 2018).

#### E3-ligases that activate NLRP3

Few E3-ligases have been linked to activation NLRP3; amongst them, Pellino2 was described to induce Lys63 ubiquitylation of NLRP3 following chronic LPS stimulation. The ubiquitylation activity of Pellino2 occurs through FHA and RING-like domains where Pellino2 promotes the ubiquitination of NLRP3. In this paper the authors postulated that there is counter regulatory relationship between Pellino2 and IL-1 receptor associated kinase 1(IRAK1), which explains the negative regulatory function of IRAK1 in NLRP3 inflammasome activation (Humphries et al., 2018). In contrast, other researchers have published that IRAK1 is required for the rapid activation of the NLRP3 inflammasome (Fernandes-Alnemri et al., 2013; Lin et al., 2014). A recent paper showed that HUWEI E3 ligase mediates Lys27-linked poly-ubiquitination of NLRP3 to induce its activation. The BH3 domain of the E3 ubiquitin ligase HUWE1 was found to interact with NLRP3 through the NACHT domain (Guo et al., 2020). Finally, an E2 conjugating enzyme (Ubc13) has been implicated in NLRP3 ubiquitylation and activation. Ubc13 interacts with NLRP3 and mediates its Lys63 Poly-ubiquitylation at residues Lys565 and Lys687 (Ni et al., 2021).

#### 4.1.3 NLRP6

The NLRP6 inflammasome is crucial in regulating inflammation and host defense against microorganisms in the intestine. In this context, it has been described that Cyld is able to de-ubiquitylates NLRP6 in a Lys63 poly-ubiquitin chain dependent manner and this has an impact on NLRP6-ASC complex formation and production and maturation of IL-18. (Mukherjee et al., 2020).

#### 4.1.4 NLRP7

The function of NLRP7 is less understood than other NLRs. It has been linked to innate immune signaling, but its precise function is still controversial. Only 3 potential lysines have been identified by massive screening efforts, that potentially could be ubiquitylated in NLRP7 although none of these sites have been validated (Akimov et al., 2018) (Table 2). The first evidence between NLRP7 and ubiquitin pathway was published in 2017, where Bednash and colleagues described that the DUB STAM-binding protein (STAMBP) was able to inhibit NLRP7 activity through deubiquitylation of poly-ubiquitin chains conjugated to Lys288 and Lys290 residues (Bednash et al., 2017). The authors postulated that NLRP7 is constitutively ubiquitylated and recruited to the endolysosome for degradation. STAMBP increases NLRP7 protein levels by impairing its trafficking to lysosomes. USP2, Trabid, and STAMBP were identified as potential DUBs of the process but considering the promiscuity of USP2 and ubiquitin chain specificity of Trabid and STAMBP for Lys63, the authors refer that the modification of NLRP7 is by this type of chain, although further experiments were only done with the DUB STAMBP. So, it would be interesting to explore Trabid role in NLRP7 context (Bednash et al., 2017). USP10 has been reported to deubiquitylate NLRP7 at Lys379 to enhance its stability and expression to promote tumor progression and tumor associated M2 macrophage polarization in colorectal cancer (Li B. et al., 2021).

#### 4.1.5 NLRP12

NLRP12 plays an essential role as a potent suppressor of inflammation. NLRP12 has been linked to the E3-ubiquitin ligase TRIM25, this TRIM25 ubiquitination by poly- ubiquitin Lys63 chains, and the activation of RIG-I. NLRP12 is also responsible for the Lys48 poly ubiquitylation of RIG-I through the E3-ligase RNF125 (Chen et al., 2019).

#### 4.1.6 NLRP14

NLRP14 has been described to have a role in spermatogenesis and inflammation (Yin et al., 2020). NLRP14 interacted physically with the nucleic acid sensing pathway and targeted TBK1 (TANK binding kinase 1) for ubiquitination and degradation. In this publication, the authors demonstrated that this ubiquitylation degradation is independent of Lys48 and Lys63 poly-ubiquitin chains. All these discoveries could have an impact on fertilization (Abe et al., 2017). Several annotated motifs of this receptor can be found PhosphositePlus database (Table 2).

#### 4.1.7 NLRC4

The BH3 domain of the E3 ubiquitin ligase HUWE1 was found to interact with NLRC4 through the NACHT domain. HUWE1 mediated the Lys27-linked polyubiquitination of NLRC4 which activates this inflammasome (Guo et al., 2020).

Recently, it has been demonstrated that HERC2 another E3-ligase is able to interact with NLRC4 and mediates its ubiquitylation and this is possibly regulated by the Long non-coding RNA (LncRNA-Fendrr) (Wang L.-Q. et al., 2021).

#### 4.1.8 Rest of Described NOD-Like Receptors

NLRP2 has been attributed in the literature to different roles, including an interaction with TBK1, which negatively impacts antiviral immunity (Yang et al., 2018). Despite several annotated motif in the PhosphositePlus database, not a single ubiquitylation has been identified as of February 2022 (see Table 2) no single one has been validated yet.

NLRP5 has a role in oocyte implantation (Fernandes et al., 2012) and female infertility (Huang et al., 2022). The three remaining NLRs; NLRP8, NLRP9, NLRP10 and NLRP13 have described functions in innate immunity and inflammation. For all of them, according to our research for this review, no ubiquitylation has been described experimentally or by in silico prediction software with well described ubiquitylation motifs.

### 4.2 Absent in Melanoma 2

In 2011 Liu et al. (2016) described tripartite motif 11 (TRIM11) as a key negative regulator of the AIM2 inflammasome. TRIM11 is an E3-ligase that induces its auto-polyubiquitination at Lys458 after binding to AIM2 and promotes AIM2 degradation. More recently, USP21 has been identified as a DUB that deubiquitylates AIM2 and induce its stability (Hong et al., 2021). The authors also demonstrated that USP21 is essential in AIM2 inflammasome assembly and absence of USP21 does not affect binding of AIM2 to DNA but impairs AIM2-ASC assembly (Hong et al., 2021).

Moreover, Lys39 and Lys235 were identified as a new ubiquitylation sites in a mass spectrometry screening (PhosphositePlus). The identification of E3-ligase(s), which mediates ubiquitylation of Lys39 and Lys235 could shed light on the AIM2 inflammasome regulation (Hornbeck et al., 2012; Hornbeck et al., 2019).

Finally, the E3-ligase HUWE1 has been described as an interactor of AIM2. The BH3 domain of HUWE1 was important for its interaction with the HIN domain of AIM2. This interaction leads to the Lys27-linked polyubiquitination of AIM2 leading to the assembly of this inflammasome (Guo et al., 2020).

### 4.3 ASC

ASC is post-translationally modified by phosphorylation and ubiquitylation, although for the latter fewer information are available. Guan et al. (2015) described that TNFR-associated factor 3 (TRAF3) was the E3-ligase responsible for ASC ubiquitylation at Lys174, and they demonstrated that the process is essential in ASC speck formation (Guan et al., 2015). Activation of the AIM2 inflammasome with poly (dA:dT) results in Lys63-linked polyubiquitination of ASC in macrophages mediated by p62 (Shi et al., 2012). Whereas the linear ubiquitylation of ASC can be mediated by LUBAC ubiquitin complex to mediates NLRP3/ASC assembly (Rodgers et al., 2014). More recently, Zhang and colleagues discovered the modification of ASC at Lys55 by Peli1 (Zhang et al., 2021). In this case the ubiquitin chain formed is Lys-63 facilitates ASC/NLRP3 interaction and ASC oligomerization and inflammasome activation. Moreover, TRAF6 have been linked to ASC ubiquitylation (Chiu et al., 2016).

On the other hand, USP50 has been described as a DUB able to deubiquitylate Lys-63 ubiquitin chains from ASC (Lee et al., 2017). Apart of these examples, not much more is found in the literature about ASC ubiquitylation, although some PTM sites are described on PhosphositePlus database because of the massive screening studies (all summarized in Table 2).

### 4.4 Caspase-1

Despite its tremendous importance in the inflammasome complex no ubiquitin PTMs have been demonstrated on Caspase-1 protein. On the PhosphositePlus database several lysines in the Caspase-1 sequence have been postulated to be ubiquitylated, including Lys37, Lys44, Lys53, Lys134, Lys148, Lys158, Lys204, Lys268, Lys274, Lys278, Lys319, Lys320, and Lys325 (see Table 2). All of them are a result of massive mass spectrometry analysis including the more recent one carried out by Akimov et al. (2018), but so far, no biochemical experiments have been published to prove the ubiquitylation of these sites or potential functional effects. Further investigations are needed to demonstrate the regulation of Caspase-1 by ubiquitin PTMs. On the other hand, several publications indicate the potential role of multiple DUBs in Caspase-1 activity regulation (Lopez-Castejon et al., 2013), but no direct ubiquitylation of Caspase-1 has been proven to date.

### 4.5 Caspase-4

Reported as an inflammatory caspase, (Martinon and Tschopp, 2007), the only reported ubiquitin modifications or residues of Caspase-4 can be found on PhosphositePlus. Yet again, validation of Lys87, Lys107, and Lys129 is required to understand the significance of this discovery (Table 2).

### 4.6 IL-1β

Interleukin-1α and -β are key molecules regulated by inflammasome effector proteins. Several years ago, IL-1α and -β were reported to be ubiquitylated and degraded *via* proteasome (Ainscough et al., 2014). More recently, a precursor of IL-1β has been demonstrated to be ubiquitylated at Lys133 consisting of mixed Ly11, 48 and 63 poly-Ub chains, promoting its proteasomal degradation. Consequently, Lys133Arg mutants have increased level of IL-1b percussor, the mutation abrogated IL-1β processing and secretion (Vijayaraj et al., 2021).

### 4.7 Gasdermin D

Inflammatory caspases cleave the pore-forming protein called gasdermin D (GSDMD) to active GSDMD amino (N)-terminus to bind to the cell membrane to induce a highly inflammatory form of programmed cell death called pyroptosis (Kayagaki et al., 2015). PhosphositePlus has reported several potential lysine residues ubiquitylated in GSDMD (Wagner et al., 2011; Udeshi et al., 2013) (Table 2). Two of these sites Lys203 and Lys204 has been reported to be ubiquitylated by SYVN1, an E3-ligase that mediates K27 polyubiquitylation of GSDMD and promotes its activation (Shi et al., 2022).

Other authors have recently described that *Shigella* ubiquitin ligase IpaH7.8 is able to ubiquitylate GSDMD and promotes its degradation *via* proteasome in humans but not in rodents (Luchetti et al., 2021).

Another paper also refers to the ubiquitylation of GSDMD mediated by an environmental toxicant, sodium arsenite (NaAsO2). Their data showed reduced GSDMD Lys48 and Lys63 polyubiquitylation inhibiting its degradation *via* proteasome to promote pyroptosis. These findings could have an impact on prevention and treatment of type 2 diabetes (Zhu et al., 2022).

The validated residues that are ubiquitylated in inflammasome components so far are represented in (Figures 2A,B). Future studies are needed to provide a deeper understanding of:

1) upstream E3-ligases and corresponding DUBs
2) temporal and spatial control of ubiquitylation events.
3) functional characterization of identified ubiquitylation sites, in particular beyond the NLRP3 inflammasome.

## 5 Inflammasome Components SUMOylation

Little is known about the ubiquitylation process regarding inflammasome components, even less data is available for SUMOylation. SUMOylation is similar to the ubiquitin cascade with 3 coupled enzymes E1, E2, and E3 SUMOylation enzymes where a small ubiquitin-related modifier (SUMO) protein is transferred to the substrate (Figure 1). The majority of functions of this PTM process are to establish non-covalent protein-protein interactions between SUMOylated substrates and their binding partners (Lascorz et al., 2021). This process has been linked to the most studied inflammasome component NLRP3, but how this PTM could regulate other inflammasome components is still an area of unexplored biology.

In particular, NLRP3 is SUMOylated by the SUMO E3-ligase MAPL which impairs its activation (Barry et al., 2018). In the same publication, SENP6 and SENP7 are described as SUMO-specific proteases that revert this process and increase NLRP3 inflammasome activation. More recently, Shao et al. (2020) described how SUMO-conjugating enzyme (UBC9) is able to SUMOylates NLRP3 at the Lys204 site. In addition, this study described SENP3 to mediate the reversible process (Table 3). In the same line, it has been described that knockdown of SENP7 decreases NLRP3 inflammasome activity (Li et al., 2020). Interestingly, SENP3 action has a negative effect on speck formation and inflammasome activation in contrast to what SENP6 and SENP7 function (Shao et al., 2020). Aligned with UBC9 data, Qin et al. (2021) have described that E3 SUMO ligase tripartite motif-containing protein 28 (TRIM28) is an enhancer of NLRP3 inflammasome activation by facilitating NLRP3 expression. TRIM28 binds NLRP3, promotes SUMO1, SUMO2, and SUMO3 modification of NLRP3, and impairs NLRP3 ubiquitination degradation *via* proteasome.

**TABLE 3:** Summary of human inflammasome other less common PTMs. In orange are represented residues non validated by recorded in PhosphositePlus whereas in green are validated residues.

Protein	Acetylation	Methylation	SUMOylation	Reference	Inflammasome modulation
AIM2	Lys71, Lys79, Lys85, Lys160	Lys64		Akimov et al. (2018)	Unknown
NALP1 (NLRP1)	Lys1428, Lys1439			Akimov et al. (2018)	Unknown
NLRP2				Akimov et al. (2018)	Unknown
NLRP3	Lys21, Lys22 and Lys24 (SIRT2)		Lys204 (SUMO1/SENP3)	(He et al., 2020; Shao et al., 2020)	Activation
NALP4 (NLRP4)	Lys611			Akimov et al. (2018)	Unknown
NLRP5		Arg20		Akimov et al. (2018)	Unknown
NLRP6	Lys376			Akimov et al. (2018)	Unknown
NLRP7	Lys189, Lys855, Lys847			Akimov et al. (2018)	Unknown
NLRP8					
NLRP9	Lys303, Lys304, Lys314, Lys316			Akimov et al. (2018)	Unknown
NALP10 (NLRP10)	Lys332, Lys605			Akimov et al. (2018)	Unknown
NLRP11			Lys84	Akimov et al. (2018)	Unknown
NLRP12	Lys1020			Akimov et al. (2018)	Unknown
NLRP13	Lys562, Lys567				
NLRP14		Arg34, Arg444		Akimov et al. (2018)	Unknown
NLRC4	Lys71, Lys72			Guan et al. (2021)	Unknown
Caspase-1					
Caspase-4		Lys49, Lys53		Akimov et al. (2018)	Unknown

The only validated residue that is SUMOylated in NLRP3 so far are represented in (Figure 2A). Future studies are needed to provide a deeper understanding of how SUMOylation regulates the different inflammasome components.

## 6 Other Less Known Post-Translational Modifications

### 6.1 Acetylation

Lysine acetylation is a PTM mediated by lysine acetyltransferases (KATs). This is a reversible process catalysed by lysine deacetylases (KDACs) (Gil et al., 2017) (Figure 1). Inflammasome-induced ASC and NLRP3 colocalization by microtubule re-arrangement via acetylation of *α*-tubulin due to sirtuin 2 (SIRT2) deacetylase inactivation was reported (Misawa et al., 2013), but acetylation of inflammasome components were not assessed in this paper. The role of microtubules in NLRP3 inflammasome assembly has been challenged, although activation of pyrin inflammasome appears critically dependent on this process (Park et al., 2016; Van Gorp et al., 2016). A recent study demonstrated that acetylation of NLRP3 at Lys21, Lys22, and Lys24 (in mouse, Lys23, Lys24, and Lys26 in human NLRP3) promotes activation of the NLRP3 inflammasome, which is controlled by the NAD-dependent protein deacetylase sirtuin-2 (SIRT2). LPS and ATP were shown to promote the acetylation level on NLRP3, and in turn increase the production of IL-1β, which was abrogated by Lys21/22/24Arg mutant. The role of SIRT2-NLRP3 axis has also been implicated in the aging-associated chronic inflammation and insulin resistance (He et al., 2020). SIRT3 deficiency leads to significantly impaired NLRC4 inflammasome activation, ASC oligomerization, and pyroptosis both *in vitro* and *in vivo*. SIRT3 interacts with and deacetylates NLRC4 at Lys71 or Lys272 to promote its activation (Guan et al., 2021). However, SIRT3 is dispensable for NLRP3 and AIM2 inflammasome.

### 6.2 ADP-Ribosylation

ADP-ribosylation is a reversible process that mediate the addition of one or more ADP-ribose to a protein (Cohen and Chang, 2018) (Figure 1). Many pathogen-derived molecules have been reported to assemble the NLRP3 inflammasome complex. It was shown that a *Mycoplasma pneumonia-*derived toxin and designated community-acquired respiratory distress syndrome (CARDS) toxin, which interact with and activates NLRP3 inflammasome, dependent on its ADP-ribosyltransferase activity. Upon stimulation by these toxins, NLRP3 was found to be ADP-ribosylated, suggesting that ADP-ribosylation of NLRP3 is a functional PTM for its positive regulation, but further investigation is required to understand the molecular basis of inflammasome activation (Bose et al., 2014).

### 6.3 Nitrosylation

Nitrosylation is a chemical process that add a NO (nitric oxide) group to a thiol (S-H) group of a protein (Mannick and Schonhoff, 2004) (Figure 1). Endogenous nitrous oxide (NO) has been shown to regulate NLRP3 inflammasome activation by either IFN-β pre-treatment or chronic LPS stimulation. Moreover, S-nitroso-N-acetylpenicillamine (SNAP), an NO donor, markedly inhibited NLRP3 inflammasome activation, S-nitrosylation of NLRP3 was detected in macrophages treated with SNAP, and this modification may account for the NO mediated mechanism controlling inflammasome activation. Further investigation is required to understand the mechanism by which nitrosylation regulate NLRP3 inflammasome activation is not understood (Hernandez-Cuellar et al., 2012).

### 6.4 Prenylation

Prenylation is a post-translational modification involving transfer of farnesyl or geranylgeranyl lipid groups on characteristic C-terminal CAAX motifs of proteins (Figure 1). Prenylation of small GTPases of Rab, Ras and Rho families is important for their cell membrane anchorage and for a variety of critical cell processes, including membrane trafficking, integrin signaling and apoptosis (Wang and Casey, 2016). A link between inflammasome activation and defective protein prenylation leading to cytosolic accumulation of unprenylated Rab and other small GTPases was identified as the underlying cause of auto-inflammatory disease resulting from recessive hippomorphic mutation in the mevalonate kinase gene. Defective activity of mevalonate kinase, a key component of mevalonate-cholesterol biosynthesis pathway, leading to decreased production of isoprenoid lipids and lack of protein prenylation (Mandey et al., 2006; Munoz et al., 2017). Studies from two different laboratories suggested that loss of K-Ras (Akula et al., 2016) or Rho GTPase (Park et al., 2016) prenylation led to pyrin inflammasome activation in murine macrophages. In contrast, a recent study, using human monocytic cell line (THP-1) or primary PBMC, has implicated NLRP3 inflammasome as the key driver of IL-1β release in cells with defective protein prenylation (Skinner et al., 2019). The discrepancies between these studies may be explained by different cell types used in these studies and emphasizes the importance of using relevant cell type for dissecting and elucidating signaling networks involved in the pathogenesis of human diseases.

### 6.5 Citrullination

Citrullination of proteins is a process in which arginine residue of a protein is converted to citrulline by deamination, which is catalyzed by peptidyl arginine deiminases (PADs) (Figure 1). This process coverts positively charged arginine to citrulline, which leads to increase in the hydrophobicity of proteins, changes in protein folding, and thereby affecting their structure and function. These citrullinated proteins are thought to act as neoantigens for the immune system to mount an immune response to produce antibodies against them causing auto-immune disorders [reviewed in (Ciesielski et al., 2022)]. However, the cellular and molecular basis of protein citrullination in autoimmune disease pathogenesis is not fully understood. The PADs have been suggested to play a role in NETosis, a process implicated in several autoimmune diseases including RA and lupus. Recently, PAD4 was reported to promote NETosis by regulating NLRP3 inflammasome activation through post-transcriptional regulation of ASC and NLRP3 levels (Munzer et al., 2021). PAD2 and PAD4 have also been shown to play a role in NLRP3 inflammasome activation in macrophages (Mishra et al., 2019). Future studies are warranted to elucidate the link between protein citrullination and NLRP3 inflammasome activation.

## 7 Role of Inflammasomes in Disease

Since the discovery of the NLRP3 inflammasome by the team of Jürg Tschopp at the University of Lausanne in 2002 (Martinon et al., 2002), much progress was made in the field to identify additional inflammasome sensors, elucidate molecular mechanism controlling inflammasome activity for example through PTMs, subcellular localization and protein-protein interactions, as well as shed light into the downstream effects of inflammasome activity through release of inflammatory mediators (DAMPs and the cytokines IL-1β and IL-18) and inflammatory cell death.

Unsurprisingly given its central role in controlling important biological effects, inflammasome components have been increasingly linked either by genetic associations, changes in expression of inflammasome components, including the effector molecules such as IL-1β and IL-18 as well as *in vitro* and *in vivo* experimentation, with multiple diseases (for example neurological disorders, metabolic diseases, autoimmune, systemic autoinflammatory diseases, respiratory diseases and cancer) (Pinkerton et al., 2017; Yi, 2018; Fernandes et al., 2020; Tartey and Kanneganti, 2020; Li Y. et al., 2021).

A well-established causal relationship between gain of function mutations of NLRP3, resulting in excessive inflammasome activation with subsequent overproduction of IL-1β, and disease can be found for autoinflammatory conditions called cryopyrin-associated periodic syndromes (CAPS) (Caseley et al., 2020; Welzel and Kuemmerle-Deschner, 2021). CAPS belongs to the category of systemic autoinflammatory diseases (SAID), which are a group of rare inherited conditions characterized by a dysregulation of the immune system and associated with recurrent episodes of fever, systemic inflammation, autoimmunity and immunodeficiency (Broderick et al., 2015; Pathak et al., 2017). CAPS encompasses a spectrum of three clinically overlapping autoinflammatory syndromes with differing degree of severity: familial cold autoinflammatory syndrome (FCAS, formerly termed familial cold-induced urticaria), the Muckle–Wells syndrome (MWS) and neonatal-onset multisystem inflammatory disease (NOMID, also called chronic infantile neurologic cutaneous and articular syndrome or CINCA) (Welzel and Kuemmerle-Deschner, 2021). Current therapeutic options include anti-IL1 therapy drugs such as the recombinant IL-1RA anakinra, the neutralizing IL-1β antibody canakinumab, the soluble decoy IL-1 receptor rilonacept (Jesus and Goldbach-Mansky, 2014; ter Haar et al., 2015). However, inflammasome-dependent, but IL-1β-independent inflammatory cell death called pyroptosis that leads to the release of DAMPs to induce more inflammation has been described to be of importance in the pathology of CAPS (Brydges et al., 2013). Targeting directly NLRP3 or a different, but essential component of its inflammasome could provide a better therapeutic opportunity. While there are currently no inhibitors of ASC in advanced clinical development, Caspase-1 inhibitors such as Belnacasan (VX-765) have not been tested in clinical trials for CAPS, perhaps due to its on-target side effects. A highly selective inhibitor of the NLRP3 inflammasome called MCC950 has been discovered by Coll and colleagues (Coll et al., 2015). MCC950 blocks NLRP3-dependent inflammasome activation at nanomolar concentrations, with no effect on NLRC4, NLRP1 or AIM2 inflammasomes. MCC950 was efficacious in a mouse model of CAPS, whereas IL-1β blockade alone did not (Coll et al., 2015). Further development of NLRP3 selective inhibitors is needed that offer the opportunity to stop IL-1β-dependent and -independent morbidities in CAPS patients.

### 7.1 Disease-Associated Mutations Affecting Post-Translational Modifications of Inflammasome Components

Very little is known how gain- or loss-of function mutations affect PTMs of inflammasome components. A few examples how mutations identified in CAPS patients’ crosstalk with PTMs of NLRP3 have been published:

PKA phosphorylation of NLRP3 Ser295 inhibited the NLRP3 ATPase activity, which is required for assembly of NLRP3-ASC complexes, resulting in NLRP3 inflammasome inhibition (Mortimer et al., 2016). A cluster of mutations in NLRP3-encoding residues in proximity to Ser295 have been linked to CAPS. NLRP3-Ser295Ala phenocopied the human CAPS mutants, most notably Asp305Gly (Mortimer et al., 2016). For example, the Ser295Ala and Asp305Gly mutants displayed ATPase activity after PKA incubation in contrast to with wild-type NLRP3. It would be interesting to see, if Ser295 phosphorylation is abolished in patients carrying one or more mutations from this cluster.

The Tyr861Cys mutation in NLRP3 has been reported in patients with CINCA. Phosphorylation of this site has been identified as negative regulator of NLRP3 (Spalinger et al., 2016). Mechanistic studies with the Tyr861Phe mutation of NLRP3, which like Tyr861Cys, can no longer be phosphorylated displayed an elevated level of inflammasome activation compared with wild-type NLRP3 (Spalinger et al., 2016).

The INFEVERS (INternet periodic FEVERS) database is a registry of hereditary auto-inflammatory disorders mutations (Sarrauste de Menthiere et al., 2003). Mutations found for NLRP1, NLRP3, NLRP7, NLRP12, and NLRC4 have been collected. As of April 2022, 256 sequence variants for NLRP3 have been registered and unsurprisingly, many are in close proximity of PTMs. A PTM-focused proteomics analysis of macrophages from CAPS patients could provide valuable mechanistic insights how sequence variations in NLRP3 crosstalk with activating or inhibiting PTMs.

### 7.2 Future Perspective of Inflammasome Post-Translational Modifications for Precision-Based Medicine, Diagnostics and Identification of Novel Drug Targets

While end products of inflammasomes such as circulating ASC specks and mediators such as IL-1β and IL-18 are strong signals of inflammasome activity, they don’t provide any mechanistic insights where and which of the many NLRs are activated. Furthermore, antibody-based methods used to detect IL-1β and IL-18 might not be able to distinguish between the cleaved and fully active proteins and their uncleaved, less active precursors. Therefore, inflammasome activity might not correlate well with the expression levels of these two mediators *in vitro* and *in vivo*.

Post-translational modifications can regulate all aspects of inflammasome biology, from activation and inhibition, subcellular localization, their interactome and function. Monitoring PTM status could allow an assessment of inflammasomes activation status and of the upstream catalyzing enzymes. In the future, a well validated PTM has the potential to enable a precision medicine approach to identify a specific patient population that would benefit from selective inhibition of one respective inflammasome, if a pan inflammasome inhibition is not desirable. Likewise, a well characterized PTM might be an option for a target engagement biomarker for an inflammasome inhibitor.

PTMs have the potential to be used to control inflammasome activation in a specific manner. For instance, modulation of protein ubiquitylation could modulate protein stability, whereas some phosphorylation sites have been implicated in the subcellular localization (Guo et al., 2020; Bittner et al., 2021).

An intriguing, yet unproven application of well characterized inflammasome PTMs could be as diagnostic tools. Because CAPS is an extremely rare disease and patients show a heterogeneous multi-system clinical presentation, a significant delay exists between symptom onset and definitive diagnosis (Welzel and Kuemmerle-Deschner, 2021). Screening for and identification of a PTM associated with constitutively NLRP3 inflammasome activation in CAPS patients might in the future enable earlier diagnosis of their disease and thereby enable earlier therapeutic intervention to avoid morbidity and reduce mortality.

All PTMs are catalyzed by upstream enzymes, which could be drug targets by their own rights due to involvement in diseases not driven by excessive inflammasome activity. For some of these enzymes, a direct measurement of their activity, either in pre-clinical drug development or as target engagement or patient identification biomarker might prove to be too challenging. If a well characterized inflammasome PTM is indeed robustly predictive of upstream enzyme activity, this PTM could be tested as a more accessible, surrogate biomarker for the upstream drug target.

## 8 Conclusion

The identification of PTMs through proteomics approaches led to the discovery of a plethora of modified sites within inflammasome sensors, adaptor protein ASC and the effector caspases in recent years. Lagging behind is the functional characterization, in particular the contribution of individual PTMs and concerted action of multiple PTMs for inflammasomes. To further build on past discoveries and improve our knowledge in the future we propose:

• Development of PTM detection tools (for example PTM site-specific antibodies) that provide sufficient sensitivity and robustness to enable pre-clinical research
• Establish a deep understanding of temporal and spatial expression of PTMs
• Functional characterization of PTMs, ideally utilizing CRISPR/Cas9 gene editing to generate knock-in models
• Systematic exploration of upstream enzymes controlling PTMs, for example through validated small molecule inhibitor libraries, siRNA libraries or CRISPR/Cas9 genome editing approaches
• Translational of PTMs across species and in appropriate human biological samples from patients.
